# Association of insulin resistance indicators with hepatic steatosis and fibrosis in patients with metabolic syndrome

**DOI:** 10.1186/s12876-023-03095-6

**Published:** 2024-01-09

**Authors:** Tzu-chia Kuo, Yang-bor Lu, Chieh-lun Yang, Bin Wang, Lin-xin Chen, Ching-ping Su

**Affiliations:** 1 Department of Chinese Medicine, Xiamen Chang Gung Hospital, No.123 Xiafei Road, Haicang District, Xiamen, 361022 Fujian China; 2 Department of Digestive Diseases, Xiamen Chang Gung Hospital, Xiamen, 361022 Fujian China; 3 Department of Nephrology, Xiamen Chang Gung Hospital, Xiamen, 361022 Fujian China

**Keywords:** TyG-WHtR, Hepatic steatosis, Hepatic fibrosis, Metabolic syndrome, NHANES

## Abstract

**Background:**

To investigate the association of four insulin resistance (IR) indicators with hepatic steatosis and fibrosis in patients with metabolic syndrome (MetS), as well as to compare the diagnostic value of these indicators in identifying hepatic steatosis and fibrosis in individuals with MetS.

**Methods:**

This cross-sectional study used the data from the National Health and Nutrition Examination Survey 2017–2018. IR indicators included homeostasis model assessment of IR (HOMA-IR), triglyceride/glucose (TyG) index, triglyceride glucose-waist-to-height ratio (TyG-WHtR), and metabolic score for IR (METS-IR). The main endpoints of this study were hepatic steatosis and hepatic fibrosis. Weighted univariate and multivariate logistic regression models were employed to evaluate the association between four IR indicators and both hepatic steatosis, hepatic fibrosis. The efficacy of various IR indicators in the detection of hepatic steatosis and hepatic fibrosis were assessed using receiver operating characteristics curve (ROC).

**Results:**

A total of 876 participants with MetS were enrolled. Among the participants, hepatic steatosis was observed in 587 MetS individuals, while hepatic fibrosis was identified in 151 MetS individuals. In multivariate logistic regression model, HOMA-IR, TyG, TyG-WHtR, and METS-IR were related to the increased odd of hepatic steatosis. Additionally, HOMA-IR, TyG-WHtR, and METS-IR were associated with increased odd of hepatic fibrosis. According to the ROC analysis, the area under the curve (AUC) of the TyG-WHtR (AUC = 0.705, 95%CI: 0.668–0.743) was higher than HOMA-IR (AUC = 0.693, 95%CI: 0.656–0.730), TyG (AUC = 0.627, 95%CI: 0.587–0.666), and METS-IR (AUC = 0.685, 95%CI: 0.648–0.722) for identifying hepatic steatosis of MetS patients. Likewise, TyG-WHtR was also higher than HOMA-IR, TyG, and METS-IR for identifying hepatic fibrosis of MetS patients.

**Conclusion:**

HOMA-IR, TyG-WHtR, and METS-IR may be associated with the risk of hepatic steatosis and fibrosis among the U.S. adult population with MetS. In addition, TyG-WHtR may have a good predictive value for hepatic steatosis and hepatic fibrosis.

**Supplementary Information:**

The online version contains supplementary material available at 10.1186/s12876-023-03095-6.

## Background

Hepatic steatosis is characterized by the excessive accumulation of fat in the liver, and hepatic fibrosis involves abnormal protein deposition within the extracellular matrix [1]. Hepatic steatosis and hepatic fibrosis were recognized as two primary manifestations of non-alcoholic fatty liver disease (NAFLD) [2], and their global prevalence remains high, resulting in a huge disease burden [3, 4]. Individuals diagnosed with metabolic syndrome (MetS) often experience a significant prevalence of hepatic steatosis and hepatic fibrosis, with hepatic steatosis being recognized as one of the manifestations of metabolic syndrome in the liver [5].

In MetS patients, the presence of insulin resistance (IR) is a significant characteristic that can potentially impact the progression of the disease [6, 7]. Homeostasis model assessment of IR (HOMA-IR) [8], triglyceride/glucose (TyG) index [9], triglyceride glucose-waist-to-height ratio (TyG-WHtR) [10], and metabolic score for IR (METS-IR) [11] were considered as indicators reflecting IR. IR has been previously demonstrated to be associated with hepatic steatosis in the general population [10, 12] and hepatic fibrosis in patients diagnosed with NAFLD [13, 14]. However, there is still limited evidence about the association of IR and the risk of hepatic steatosis and hepatic fibrosis in patients who have developed MetS. In El-Sehrawy’s study, it was observed that premenopausal women diagnosed with MetS exhibited a higher HOMA-IR than healthy controls, and HOMA-IR was also associated with advanced NAFLD grade [15]. However, the clinical utility of HOMA-IR, as an improtant indicator for IR, is limited by the complexity associated with directly measuring fasting insulin levels [16]. Additionally, the findings of one study demonstrated that the METS-IR exhibited significantly superior predictive ability for advanced liver fibrosis in patients with NAFLD compared to both the TyG index and HOMA-IR [17]. To date, the value of various indicators reflecting IR in the identification of hepatic steatosis and hepatic fibrosis in patients with MetS remains unclear.

As a result, our study aimed to investigate the association between four indicators reflecting IR (HOMA-IR, TyG index, TyG-WHtR, and METS-IR) and hepatic steatosis and fibrosis in patients with MetS, as well as to compare the diagnostic value of these indicators in identifying hepatic steatosis and fibrosis in individuals with MetS, which providing a convenient tool for screening hepatic steatosis and fibrosis risk among patients with MetS.

## Methods

### Study population

The National Health and Nutrition Examination Survey (NHANES) is a nationally representative survey using a complex, stratified, multistage probability sampling design method [18]. Through interviews and physical examinations, demographics, dietary, socioeconomic, and health-related information are collected [18]. The requirement of ethical approval for this was waived by the Institutional Review Board of Xiamen Chang Gung Hospital, because the data was accessed from NHANES (a publicly available database). The need for written informed consent was waived by the Institutional Review Board of Xiamen Chang Gung Hospital due to retrospective nature of the study. All methods were performed in accordance with the relevant guidelines and regulations.

**Fig. 1:**
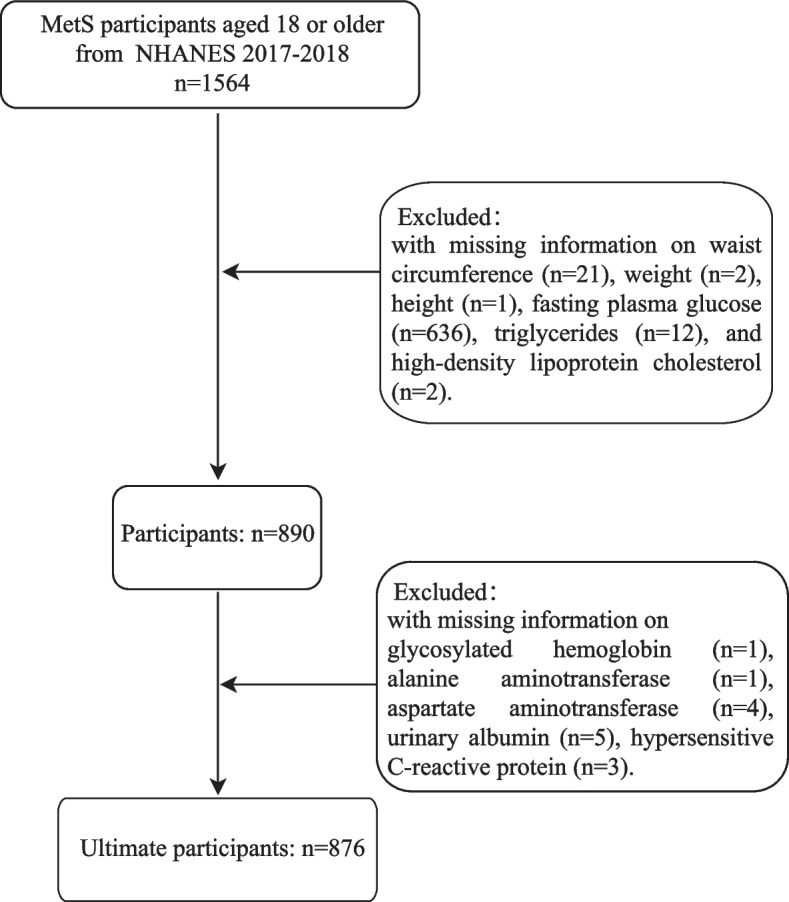
The flowchart of patient selection

In this cross-sectional study, we used the data from the NHANES database 2017–2018. Initially, participants aged 18 or older who were diagnosed with MetS were included (*n* = 1564). MetS was defined using harmonized criteria-fulfillment of a minimum of three out of the following five criteria [18]: (1) waist circumference (WC) ≥102 cm for men and ≥ 88 cm for women; (2) high blood pressure [systolic blood pressure (SBP) ≥130 mmHg, diastolic blood pressure (DBP) ≥85 mmHg, or use of blood pressure medication]; (3) triglycerides ≥1.7 mmol/L; (4) low high-density lipoprotein cholesterol (< 1.03 mmol/L in men and < 1.29 mmol/L in women); (5) fasting glucose ≥5.6 mmol/L, or with type 2 diabetes. Of these participants, we excluded some participants with missing information on waist circumference (*n* = 21), weight (*n* = 2), height (*n* = 1), fasting plasma glucose (FPG, *n* = 636), triglycerides (TG, *n* = 12), high-density lipoprotein cholesterol (HDL-C, *n* = 2), glycosylated hemoglobin (*n* = 1), alanine aminotransferase (ALT, *n* = 1), aspartate aminotransferase (AST, *n* = 4), urinary albumin (ALB, *n* = 5), hypersensitive C-reactive protein (hs-CRP, *n* = 3). Eventually, 876 participants were enrolled in the study (Fig. 1).

### Exposure variable

Indicators reflecting IR included HOMA-IR, TyG index, TyG-WHtR, and METS-IR in this study. These indicators were calculated as follows [17, 19, 20]:

1. HOMA-IR = [fasting serum insulin (μU/mL) × FPG (mg/dL)/405];
2. TyG = ln [fasting serum TG (mg/dL) × FPG (mg/dL)/2];
3. TyG-WHtR = TyG index × waist-to-height ratio (WHtR);
4. METS-IR = ln [2 × FPG (mg/dL) + fasting serum TG (mg/dL)] × body mass index (BMI, kg/m^2^)/ln [HDL-C (mg/dL)];

Each indicator reflecting IR was categorized into three groups according to the tertiles.

### Outcome variable

The main endpoints of this study were hepatic steatosis and hepatic fibrosis. In the NHANES 2017–2018 cycle, participants were assessed for Vibration Controlled Transient Elastography (VCTE) using the FibroScan Model 502 V2 Touch (Echosens, Paris, France), which was equipped with medium (M) and extra-large (XL) probes. Controlled attenuation parameter (CAP) and liver stiffness measurement (LSM) were used to assess hepatic steatosis and fibrosis, respectively [21]. CAP ≥274 dB/m was defined in this study as having hepatic steatosis; participants with CAP ≥274 and < 290 dB/m were defined as group S1 (*n* = 95), CAP ≥290 and < 302 dB/m were defined as group S2 (*n* = 69), and CAP ≥302 dB/m were defined as the group S3 (*n* = 423) [21]. LSM ≥8.2 kPa was considered as the indicative of hepatic fibrosis.

### Covariates

Possible covariates included age (years), gender, race, education level, family income-to-poverty ratio (PIR), smoking status, drinking status, physical activity, SBP (mmHg), DBP (mmHg), hepatitis, chronic kidney disease (CKD), cardiovascular disease (CVD), ALT (U/L), AST (U/L), alkaline phosphatase (ALP, IU/L), gamma glutamyl transferase (GGT, IU/L), total cholesterol (TC, mg/dL), low-density lipoprotein cholesterol (LDL-C, mg/dL), hs-CRP, mg/L, platelet count (1000 cells/uL), total bilirubin (umol/L), serum creatinine (mg/dL), serum ALB (g/L), urinary ALB (mg/L), urinary creatinine (mg/dL), drug for diabetes, drug for hypertension, drug for dyslipidemia, antiviral agents, glucocorticoids, drug induce hepatic steatosis, energy, carbohydrate, protein, total fat, and vitamin E. Smoking status was classified as “never smoked” (those who had never smoked more than 100 cigarettes in their lifetime), “used to smoke and now quit” (those who had smoked at least 100 cigarettes but did not currently smoke), and “still smoking” (those who smoked at least 100 cigarettes and currently smoke some days or every day). Drinking status were categorized as no and yes by self-report. Hepatitis was defined as hepatitis B or hepatitis C. Having CKD was determined by estimated glomerular filtration rate < 60 mL/min/1.73 m^2^ or the ratio of albumin and creatinine≥30 mg/g. Having CVD was defined as having angina, heart failure, heart attack, coronary heart disease, stroke, or congestive heart failure by self-reported, or coding of cardiovascular drugs.

### Statistical analysis

The random regression interpolation method was utilized to handle missing values, and sensitivity analyses were performed on the data both before and after treatment (Supplemental Table 1). The characteristics of the study population were statistically described in both the hepatic steatosis and non-hepatic steatosis groups, as well as in the hepatic fibrosis and non-hepatic fibrosis groups. The categorical data were presented as the number of cases and the constituent ratio [n (%)]. Mean standard error [Mean (SE)] is utilized to describe the measured data. We employed weighted univariate logistic regression analysis to identify potential confounders associated with the risk of hepatic steatosis and hepatic fibrosis, respectively (Supplemental Table 2). Weighted univariate and multivariate logistic regression models were employed to evaluate the association between IR indicators and both hepatic steatosis, hepatic fibrosis. Odds ratio (OR) and 95% confidence interval (CI) was calculated. The efficacy of various IR indicators in the detection of hepatic steatosis and hepatic fibrosis were assessed using receiver operating characteristics curve (ROC). Subgroup analysis was carried out in different population based on age (< 60/≥60 years) and gender (male/female). The statistical study was carried out using the SAS 9.4 software (SAS Institute Inc., Cary, NC, USA). *P* < 0.05 was considered statistically significant.

## Results

### Basic characteristics of included participants

A total of 876 MetS participants were involved, with an average age of 52.37 (SE = 1.18) years and a gender split of 50.07% male to 49.93% female (Tables 1 and 2). Among the participants, hepatic steatosis was observed in 587 MetS individuals, while hepatic fibrosis was identified in 151 MetS individuals. As shown in Table 1, there was a significant statistical difference observed between MetS patients with and without hepatic steatosis in terms of their education level, hepatitis, CVD, ALT, drug for diabetes, drug for hypertension, protein, HOMA-IR, TyG index, TyG-WHtR, and METS-IR (all *P* < 0.05). Additionally, we also compared the basic characteristics in the hepatic fibrosis group and non-hepatic fibrosis group (Table 2). MetS participants with hepatic fibrosis had a significantly lower family PIR, proportion of people with drinking, and platelet count (*P* < 0.05) than those with non- hepatic fibrosis.

**Table 1:** Comparison of basic characteristics of people with and without hepatic steatosis

Variables	Total (*n* = 876)	Non-hepatic steatosis group (*n* = 289)	Hepatic steatosis group (*n* = 587)	*P*
Age, years, Mean (S.E)	52.37 (1.18)	51.61 (2.01)	52.75 (1.10)	0.544
Gender, n (%)				0.103
Male	407 (50.07)	109 (44.28)	298 (52.98)	
Female	469 (49.93)	180 (55.72)	289 (47.02)	
Race, n (%)				0.455
Mexican American	154 (11.52)	37 (8.13)	117 (13.22)	
Other Hispanic	96 (6.79)	31 (6.63)	65 (6.87)	
Non-Hispanic White	308 (62.25)	100 (63.26)	208 (61.75)	
Non-Hispanic Black	162 (8.86)	66 (10.20)	96 (8.19)	
Other Race	156 (10.58)	55 (11.78)	101 (9.97)	
Education level, n (%)				0.004
High school degree or less	222 (15.10)	86 (21.24)	136 (12.02)	
High school education	214 (32.53)	52 (22.83)	162 (37.41)	
High school degree or above	429 (51.71)	147 (55.16)	282 (49.98)	
Unknown	11 (0.65)	4 (0.77)	7 (0.59)	
Family PIR, Mean (S.E)	2.80 (0.10)	2.76 (0.15)	2.81 (0.14)	0.790
Drinking status, n (%)				0.871
No	116 (9.73)	48 (10.07)	68 (9.56)	
Yes	760 (90.27)	241 (89.93)	519 (90.44)	
Smoking status, n (%)				0.875
Never smoked	457 (49.28)	158 (47.95)	299 (49.95)	
Used to smoke and now quit	255 (30.88)	74 (30.90)	181 (30.86)	
Still smoking	164 (19.85)	57 (21.16)	107 (19.19)	
Physical activity, n (%)				0.708
≤ 750 MET∙min	605 (61.46)	210 (62.89)	395 (60.74)	
> 750 MET∙min	271 (38.54)	79 (37.12)	192 (39.26)	
SBP, mmHg, Mean (S.E)	131.37 (0.63)	129.69 (1.62)	132.22 (1.00)	0.290
DBP, mmHg, Mean (S.E)	76.09 (0.64)	76.44 (1.27)	75.91 (0.63)	0.700
Hepatitis, n (%)				0.025
No	797 (93.57)	254 (89.25)	543 (95.75)	
Yes	79 (6.43)	35 (10.75)	44 (4.26)	
CKD, n (%)				0.652
No	811 (95.27)	266 (94.57)	545 (95.62)	
Yes	65 (4.73)	23 (5.43)	42 (4.38)	
CVD, n (%)				0.011
No	592 (71.57)	210 (79.72)	382 (67.48)	
Yes	284 (28.43)	79 (20.28)	205 (32.52)	
ALT, U/L, Mean (S.E)	27.25 (1.04)	23.34 (1.49)	29.21 (1.15)	0.004
AST, U/L, Mean (S.E)	23.48 (0.81)	22.40 (1.63)	24.02 (0.92)	0.408
ALP, IU/L, Mean (S.E)	81.61 (0.83)	83.04 (2.41)	80.90 (1.11)	0.494
GGT, IU/L, Mean (S.E)	36.13 (1.94)	33.41 (3.41)	37.50 (2.06)	0.281
TC, mg/dL, Mean (S.E)	191.01 (3.62)	190.37 (2.84)	191.32 (4.54)	0.808
LDL-C, mg/dL, Mean (S.E)	114.49 (2.57)	113.35 (2.18)	115.06 (3.29)	0.591
Hs-CRP, mg/L, Mean (S.E)	5.22 (0.50)	5.19 (1.32)	5.23 (0.36)	0.978
Platelet count, 1000 cells/uL, Mean (S.E)	245.33 (3.66)	249.42 (4.61)	243.28 (4.37)	0.271
Total bilirubin, umol/L, Mean (S.E)	8.12 (0.24)	8.04 (0.46)	8.16 (0.29)	0.839
Serum creatinine, mg/dL, Mean (S.E)	0.87 (0.01)	0.87 (0.02)	0.87 (0.01)	0.685
Urinary creatinine, mg/dL, Mean (S.E)	139.13 (5.45)	126.81 (10.49)	145.33 (4.25)	0.086
Serum ALB, g/L, Mean (S.E)	39.79 (0.26)	39.67 (0.39)	39.86 (0.32)	0.711
Urinary ALB, mg/L, Mean (S.E)	57.03 (8.94)	53.57 (14.45)	58.77 (11.72)	0.789
Drug for diabetes, n (%)				0.028
No	634 (77.36)	227 (83.91)	407 (74.07)	
Yes	242 (22.64)	62 (16.09)	180 (25.93)	
Drug for hypertension, n (%)				0.013
No	472 (57.54)	164 (66.39)	308 (53.09)	
Yes	404 (42.46)	125 (33.62)	279 (46.91)	
Drug for dyslipidemia, n (%)				0.129
No	591 (69.83)	206 (75.00)	385 (67.23)	
Yes	285 (30.17)	83 (25.00)	202 (32.77)	
Antiviral agents, n (%)				0.986
No	874 (99.92)	288 (99.92)	586 (99.92)	
Yes	2 (0.08)	1 (0.08)	1 (0.08)	
Glucocorticoids, n (%)				0.897
No	854 (97.73)	281 (97.83)	573 (97.68)	
Yes	22 (2.27)	8 (2.17)	14 (2.32)	
Drug induce hepatic steatosis n (%)				0.129
No	859 (97.67)	279 (96.00)	580 (98.50)	
Yes	17 (2.33)	10 (4.00)	7 (1.50)	
Energy, Mean (S.E)	2220.76 (50.98)	2126.94 (75.22)	2267.91 (67.43)	0.194
Carbohydrate, Mean (S.E)	258.88 (6.21)	252.72 (7.69)	261.97 (8.27)	0.415
Protein, Mean (S.E)	83.54 (2.04)	76.39 (3.03)	87.14 (2.79)	0.030
Total fat, Mean (S.E)	91.19 (2.41)	85.05 (4.41)	94.28 (3.09)	0.126
Vitamin E, Mean (S.E)	0.67 (0.18)	0.92 (0.45)	0.55 (0.15)	0.451
METS-IR, Mean (S.E)	53.19 (1.03)	47.68 (1.09)	55.96 (0.95)	< 0.001
TyG index, Mean (S.E)	9.14 (0.04)	8.97 (0.05)	9.22 (0.06)	0.006
HOMA-IR, Mean (S.E)	6.49 (0.37)	4.32 (0.40)	7.58 (0.52)	< 0.001
TyG-WHtR, Mean (S.E)	6.05 (0.07)	5.62 (0.07)	6.27 (0.07)	< 0.001

*PIR* income-to-poverty ratio, *SBP* systolic blood pressure, *DBP* diastolic blood pressure, *CKD* chronic kidney disease, *CVD* cardiovascular disease, *ALT* alanine aminotransferase, *AST* aspartate aminotransferase, *ALP* alkaline phosphatase, *GGT* gamma glutamyl transferase, *TC* total cholesterol, *LDL-C* low-density lipoprotein cholesterol, *hs-CRP* high-sensitivity C-reactive protein, *ALB* albumin, *METS-IR* metabolic score for insulin resistance, *TyG* triglyceride/glucose, *HOMA-IR* homeostasis model assessment of IR, *TyG-WHtR* triglyceride glucose-waist-to-height ratio

**Table 2:** Comparison of basic characteristics of people with and without hepatic fibrosis

Variables	Total (*n* = 876)	Non-hepatic fibrosis group (*n* = 725)	Hepatic fibrosis group (*n* = 151)	*P*
Age, years, Mean (S.E)	52.37 (1.18)	52.11 (1.14)	53.83 (2.24)	0.379
Gender, n (%)				0.687
Male	407 (50.07)	325 (50.58)	82 (47.24)	
Female	469 (49.93)	400 (49.42)	69 (52.77)	
Race, n (%)				0.606
Mexican American	154 (11.52)	128 (11.30)	26 (12.75)	
Other Hispanic	96 (6.79)	76 (6.25)	20 (9.84)	
Non-Hispanic White	308 (62.25)	254 (62.50)	54 (60.90)	
Non-Hispanic Black	162 (8.86)	138 (9.23)	24 (6.83)	
Other Race	156 (10.58)	129 (10.73)	27 (9.69)	
Education level, n (%)				0.248
High school degree or less	222 (15.10)	191 (15.54)	31 (12.68)	
High school education	214 (32.53)	176 (31.26)	38 (39.69)	
High school degree or above	429 (51.71)	351 (52.67)	78 (46.34)	
Unknown	11 (0.65)	7 (0.53)	4 (1.30)	
Family PIR, Mean (S.E)	2.80 (0.10)	2.86 (0.11)	2.45 (0.16)	0.040
Drinking status, n (%)				0.031
No	116 (9.73)	93 (8.52)	23 (16.51)	
Yes	760 (90.27)	632 (91.48)	128 (83.49)	
Smoking status, n (%)				0.556
Never smoked	457 (49.28)	391 (50.05)	66 (44.98)	
Used to smoke and now quit	255 (30.88)	200 (29.90)	55 (36.34)	
Still smoking	164 (19.85)	134 (20.05)	30 (18.68)	
Physical activity, n (%)				0.092
≤ 750 MET∙ min	605 (61.46)	495 (59.54)	110 (72.21)	
> 750 MET∙ min	271 (38.54)	230 (40.46)	41 (27.79)	
SBP, mmHg, Mean (S.E)	131.37 (0.63)	131.38 (0.67)	131.31 (2.01)	0.974
DBP, mmHg, Mean (S.E)	76.09 (0.64)	76.75 (0.75)	72.36 (1.48)	0.023
Hepatitis, n (%)				0.345
No	797 (93.57)	663 (94.18)	134 (90.18)	
Yes	79 (6.43)	62 (5.82)	17 (9.83)	
CKD, n (%)				0.055
No	811 (95.27)	678 (95.80)	133 (92.32)	
Yes	65 (4.73)	47 (4.20)	18 (7.68)	
CVD, n (%)				0.013
No	592 (71.57)	506 (74.23)	86 (56.71)	
Yes	284 (28.43)	219 (25.77)	65 (43.29)	
ALT, U/L, Mean (S.E)	27.25 (1.04)	25.62 (0.99)	36.35 (3.05)	0.003
AST, U/L, Mean (S.E)	23.48 (0.81)	22.03 (0.73)	31.56 (3.27)	0.011
ALP, IU/L, Mean (S.E)	81.61 (0.83)	80.95 (1.10)	85.32 (3.74)	0.334
GGT, IU/L, Mean (S.E)	36.13 (1.94)	32.75 (1.57)	55.06 (6.87)	0.005
TC, mg/dL, Mean (S.E)	191.01 (3.62)	191.97 (3.35)	185.58 (6.05)	0.153
LDL-C, mg/dL, Mean (S.E)	114.49 (2.57)	115.17 (2.32)	110.62 (5.13)	0.281
Hs-CRP, mg/L, Mean (S.E)	5.22 (0.50)	4.95 (0.57)	6.71 (1.09)	0.174
Platelet count, 1000 cells/uL, Mean (S.E)	245.33 (3.66)	247.28 (4.06)	234.42 (4.97)	0.031
Total bilirubin, umol/L, Mean (S.E)	8.12 (0.24)	8.01 (0.25)	8.70 (0.37)	0.087
Serum creatinine, mg/dL, Mean (S.E)	0.87 (0.01)	0.87 (0.01)	0.87 (0.03)	0.892
Urinary creatinine, mg/dL, Mean (S.E)	139.13 (5.45)	139.34 (6.58)	137.99 (12.73)	0.933
Serum ALB, g/L, Mean (S.E)	39.79 (0.26)	39.96 (0.23)	38.85 (0.47)	0.008
Urinary ALB, mg/L, Mean (S.E)	57.03 (8.94)	45.93 (7.36)	119.26 (32.68)	0.036
Drug for diabetes, n (%)				< 0.001
No	634 (77.36)	553 (80.49)	81 (59.81)	
Yes	242 (22.64)	172 (19.51)	70 (40.19)	
Drug for hypertension, n (%)				0.329
No	472 (57.54)	409 (59.01)	63 (49.30)	
Yes	404 (42.46)	316 (40.99)	88 (50.71)	
Drug for dyslipidemia, n (%)				0.934
No	591 (69.83)	500 (69.76)	91 (70.23)	
Yes	285 (30.17)	225 (30.24)	60 (29.77)	
Antiviral agents, n (%)				
No	874 (99.92)	723 (99.91)	151 (100.00)	
Yes	2 (0.08)	2 (0.10)	0 (0.00)	
Glucocorticoids, n (%)				0.018
No	854 (97.73)	705 (97.42)	149 (99.45)	
Yes	22 (2.27)	20 (2.58)	2 (0.55)	
Drug induce hepatic steatosis n (%)				0.666
No	859 (97.67)	710 (97.81)	149 (96.84)	
Yes	17 (2.33)	15 (2.19)	2 (3.16)	
Energy, Mean (S.E)	2220.76 (50.98)	2230.40 (53.05)	2166.76 (106.88)	0.565
Carbohydrate, Mean (S.E)	258.88 (6.21)	261.61 (6.74)	243.59 (15.49)	0.304
Protein, Mean (S.E)	83.54 (2.04)	83.31 (2.41)	84.85 (5.29)	0.809
Total fat, Mean (S.E)	91.19 (2.41)	91.22 (2.66)	91.03 (4.93)	0.972
Vitamin E, Mean (S.E)	0.67 (0.18)	0.70 (0.21)	0.53 (0.28)	0.629
METS-IR, Mean (S.E)	53.19 (1.03)	51.64 (0.84)	61.89 (1.92)	< 0.001
TyG index, Mean (S.E)	9.14 (0.04)	9.11 (0.05)	9.27 (0.07)	0.097
HOMA-IR, Mean (S.E)	6.49 (0.37)	5.76 (0.30)	10.56 (1.98)	0.036
TyG-WHtR, Mean (S.E)	6.05 (0.07)	5.92 (0.06)	6.79 (0.12)	< 0.001

*PIR* income-to-poverty ratio, *SBP* systolic blood pressure, *DBP* diastolic blood pressure, *CKD* chronic kidney disease, *CVD* cardiovascular disease, *ALT* alanine aminotransferase, *AST* aspartate aminotransferase, *ALP* alkaline phosphatase, *GGT* gamma glutamyl transferase, *TC* total cholesterol, *LDL-C* low-density lipoprotein cholesterol, *hs-CRP* high-sensitivity C-reactive protein, *ALB* albumin, *METS-IR* metabolic score for insulin resistance, *TyG* triglyceride/glucose, *HOMA-IR* homeostasis model assessment of IR, *TyG-WHtR* triglyceride glucose-waist-to-height ratio

### Association between IR indicators and both hepatic steatosis, hepatic fibrosis

Table 3 showed the results of the weighted univariate and multivariate regression analyses. In adjusted model, compared to the referent (lower tertiles of HOMA-IR), both the middle (OR = 2.36, 95% CI: 1.14–4.87, *P* = 0.023) and highest tertiles (OR = 4.54, 95% CI: 2.06–10.03, *P* = 0.001) of HOMA-IR were related to the increased odd of hepatic steatosis. Similarly, after adjusting for confounding factors, we observed a significant association between the highest tertiles of TyG index (OR = 2.24, 95% CI: 1.15–4.39, *P* = 0.021), the middle (OR = 2.23, 95% CI: 1.17–4.24, *P* = 0.018) and highest tertiles (OR = 6.07, 95% CI: 3.74–9.83, *P* < 0.001) of TyG-WHtR, and the highest tertiles (OR = 5.60, 95% CI: 3.34–9.40, *P* < 0.001) of METS-IR with an elevated odd of hepatic steatosis. In addition, we further analyzed of four IR indicators and different degrees of hepatic steatosis. The reference group was formed by combining the SI group (*n* = 95) and S2 group (*n* = 69) due to the limited sample size. As presented Supplemental Table 3, we found that the middle (OR = 1.99, 95% CI: 1.25–3.19, *P* = 0.007) and highest tertiles (OR = 4.72, 95% CI: 2.75–8.10, *P* < 0.001) of METS-IR, highest tertiles of HOMA-IR (OR = 3.12, 95% CI: 1.11–8.79, *P* = 0.034), and the highest tertiles of TyG-WHtR (OR = 4.99, 95% CI: 2.35–10.59, *P* < 0.001) with an elevated risk of severe hepatic steatosis (group S3) in fully adjusted model.

**Table 3:** The association between four IR indicators and both hepatic steatosis, hepatic fibrosis

Indicators	Hepatic steatosis				Hepatic fibrosis			
	Crude Model		Adjusted Model^a^		Crude Model		Adjusted Model^b^	
	OR (95% *CI)*	*P*	OR (95% CI)	*P*	OR (95% CI)	*P*	OR (95% CI)	*P*
HOMA-IR								
< 3.11	Ref		Ref		Ref		Ref	
3.11–5.81	2.41 (1.17–4.94)	0.020	2.36 (1.14–4.87)	0.023	3.29 (1.16–9.37)	0.028	2.84 (0.95–8.50)	0.060
≥ 5.81	5.30 (2.65–10.62)	< 0.001	4.54 (2.06–10.03)	0.001	7.25 (3.34–15.76)	< 0.001	4.47 (1.87–10.66)	0.002
TyG								
< 8.90	Ref		Ref		Ref		Ref	
8.90–9.30	1.12 (0.58–2.15)	0.726	1.11 (0.61–2.02)	0.708	0.87 (0.37–2.01)	0.724	0.72 (0.33–1.57)	0.383
≥ 9.30	2.28 (1.10–4.74)	0.030	2.24 (1.15–4.39)	0.021	1.41 (0.58–3.41)	0.420	0.79 (0.30–2.09)	0.610
TyG-WHtR								
< 5.55	Ref		Ref		Ref		Ref	
5.55–6.29	2.77 (1.48–5.19)	0.003	2.23 (1.17–4.24)	0.018	2.34 (1.05–5.20)	0.039	1.53 (0.49–4.73)	0.439
≥ 6.29	7.01 (4.24–11.57)	< 0.001	6.07 (3.74–9.83)	< 0.001	11.42 (5.89–22.15)	< 0.001	9.21 (2.90–29.22)	< 0.001
METS-IR								
< 46.27	Ref		Ref		Ref		Ref	
46.27–57.04	1.49 (0.85–2.60)	0.148	1.53 (0.78–2.99)	0.200	1.05 (0.46–2.43)	0.900	1.28 (0.67–2.48)	0.428
≥ 57.04	6.27 (4.00–9.81)	< 0.001	5.60 (3.34–9.40)	< 0.001	4.61 (2.25–9.44)	< 0.001	5.04 (2.47–10.27)	< 0.001

*METS-IR* metabolic score for insulin resistance, *TyG* triglyceride/glucose, *HOMA-IR* homeostasis model assessment of IR, *TyG-WHtR* triglyceride glucose-waist-to-height ratio, *OR* odds ratio, *CI* confidence interval

Crude model: confounding variables were not adjusted

^a^Adjusted Model: education level, hepatitis, cardiovascular disease (CVD), alanine aminotransferase (ALT), drug for hypertension, and protein were adjusted

^b^ Adjusted Model: family income-to-poverty ratio, diastolic blood pressure, CVD, ALT, aspartate aminotransferase, gamma glutamyl transferase, platelet count, albumin, drug for diabetes, antiviral agents, and hepatic steatosis were adjusted

Also, we assessed the relationship of four IR indicators and hepatic fibrosis (Table 3). The highest tertiles of HOMA-IR (OR = 4.47, 95% CI: 1.87–10.66 *P* = 0.002), highest tertiles of TyG-WHtR (OR = 9.21, 95% CI: 2.90–29.22, *P* < 0.001), and the highest tertiles of METS-IR (OR = 5.04, 95% CI: 2.47–10.27, *P* < 0.001) were associated with increased odd of hepatic fibrosis.

### Diagnostic value of IR indicators in identifying hepatic steatosis and fibrosis

**Fig. 2:**
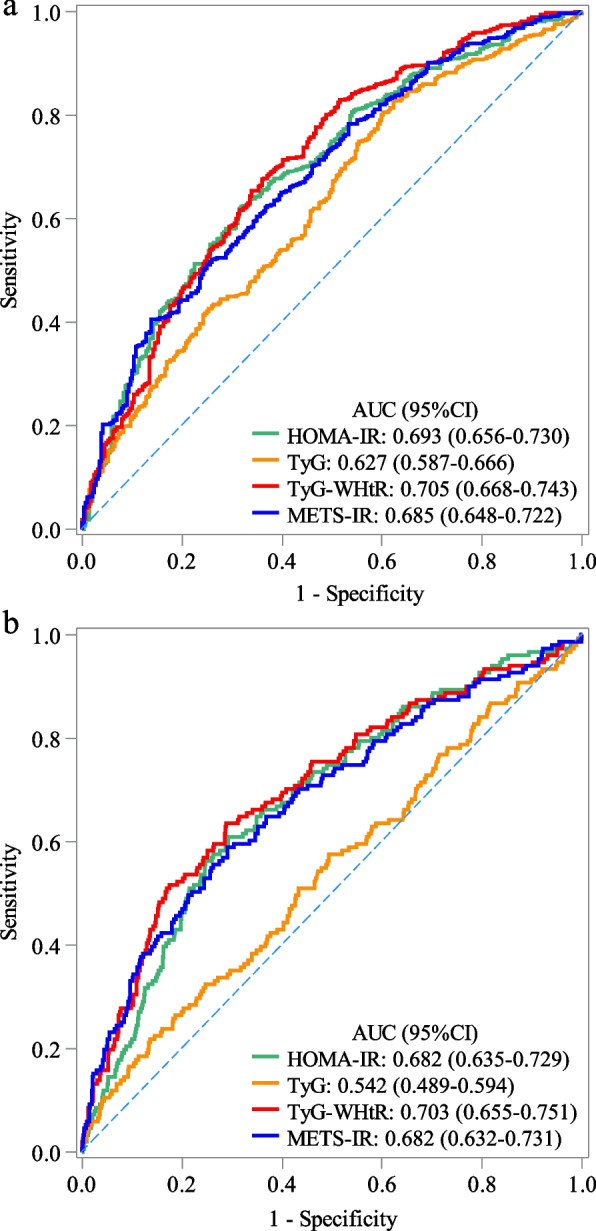
Receiver operating characteristics curves of four insulin resistance indicators to detect (**a**) hepatic steatosis and (**b**) hepatic fibrosis

The ROC curves for four IR indicators to detect hepatic steatosis and hepatic fibrosis are presented in Fig. 2a and b. According to the ROC analysis, the area under the curve (AUC) of the TyG-WHtR (Table 4; AUC = 0.705, 95%CI: 0.668–0.743) was higher than HOMA-IR (AUC = 0.693, 95%CI: 0.656–0.730), TyG index (AUC = 0.627, 95%CI: 0.587–0.666), and METS-IR (AUC = 0.685, 95%CI: 0.648–0.722). As presented in Table 4, the sensitivity, specificity, NPV, PPV, and accuracy of TyG-WHtR was 0.676 (95% CI: 0.637–0.714), 0.640 (95% CI: 0.582–0.696), 0.493 (95% CI: 0.442–0.545), 0.792 (95% CI: 0.754–0.827) and 0.664 (95% CI: 0.632–0.696) for identifying hepatic steatosis of MetS patients. Likewise, the AUC of the TyG-WHtR (AUC = 0.703, 95%CI: 0.655–0.751) was also higher than HOMA-IR (AUC = 0.682, 95%CI: 0.635–0.729), TyG index (AUC = 0.542, 95%CI: 0.489–0.594), and METS-IR (AUC = 0.682, 95%CI: 0.632–0.731). The sensitivity, specificity, NPV, PPV, and accuracy of TyG-WHtR was 0.629 (95% CI: 0.547–0.706), 0.712 (95% CI: 0.677–0.744), 0.902 (95% CI: 0.875–0.925), 0.313 (95% CI: 0.261–0.368), and 0.697 (95% CI: 0.666–0.728) for identifying hepatic fibrosis of MetS patients.

**Table 4:** Predictive performance of four IR indicators on hepatic steatosis, hepatic fibrosis

Variables	Indicators	AUC (95%CI)	Sensitivity (95%CI)	Specificity (95%CI)	NPV (95%CI)	PPV (95%CI)	Accuracy (95%CI)
**Hepatic steatosis**	HOMA-IR	0.693 (0.656–0.730)	0.618 (0.578–0.658)	0.685 (0.628–0.738)	0.469 (0.421–0.518)	0.800 (0.760–0.835)	0.640 (0.608–0.672)
	TyG	0.627 (0.587–0.666)	0.828 (0.795–0.858)	0.377 (0.321–0.436)	0.519 (0.449–0.588)	0.730 (0.694–0.763)	0.679 (0.647–0.710)
	TyG-WHtR	0.705 (0.668–0.743)	0.676 (0.637–0.714)	0.640 (0.582–0.696)	0.493 (0.442–0.545)	0.792 (0.754–0.827)	0.664 (0.632–0.696)
	METS-IR	0.685 (0.648–0.722)	0.404 (0.364–0.445)	0.862 (0.816–0.899)	0.416 (0.376–0.456)	0.856 (0.809–0.895)	0.555 (0.521–0.588)
**Hepatic fibrosis**	HOMA-IR	0.682 (0.635–0.729)	0.603 (0.520–0.681)	0.708 (0.673–0.740)	0.895 (0.867–0.919)	0.300 (0.249–0.355)	0.689 (0.658–0.720)
	TyG	0.542 (0.489–0.594)	0.576 (0.493–0.656)	0.506 (0.469–0.543)	0.852 (0.814–0.884)	0.196 (0.160–0.235)	0.518 (0.485–0.552)
	TyG-WHtR	0.703 (0.655–0.751)	0.629 (0.547–0.706)	0.712 (0.677–0.744)	0.902 (0.875–0.925)	0.313 (0.261–0.368)	0.697 (0.666–0.728)
	METS-IR	0.682 (0.632–0.731)	0.589 (0.507–0.669)	0.709 (0.674–0.742)	0.892 (0.864–0.916)	0.297 (0.246–0.352)	0.688 (0.657–0.719)

*METS-IR* metabolic score for insulin resistance, *TyG* triglyceride/glucose, *HOMA-IR* homeostasis model assessment of IR, *TyG-WHtR* triglyceride glucose-waist-to-height ratio, *AUC* the area under of curve, *CI* confidence interval, *PPV* positive predictive value, *NPV* negative predictive value

### Subgroup analysis

We did a subgroup analysis by age and gender to observe if the results were applicable to the different population (Table 5). An association between HOMA-IR, METS-IR and hepatic steatosis was observed in each subgroup, stratified by age and gender (*P* < 0.05). The age-stratified subgroup analysis revealed a statistically significant association between TyG-WHtR and hepatic steatosis in individuals aged< 60 years (OR = 6.33, 95%CI: 3.31–12.08, *P* < 0.001), while no such association was observed in those aged ≥60 years (*P* > 0.05). The relationship of TyG index and hepatic steatosis was only observed among female MetS patients (OR = 2.94, 95%CI: 1.54–5.62, *P* = 0.003).

**Table 5:** Subgroup analysis based on age (< 60/≥60 years) and gender (male/female)

Variables	Hepatic steatosis^a^		Hepatic fibrosis^b^	
	Adjusted Model		Adjusted Model	
	OR (95% CI)	*P*	OR (95% CI)	*P*
**Age < 60 years (*n*** **= 479)**				
HOMA-IR				
< 3.11	Ref		Ref	
3.11–5.81	2.08 (0.94–4.62)	0.070	3.77 (0.75–18.97)	0.100
≥ 5.81	3.74 (1.35–10.36)	0.015	5.31 (1.29–21.83)	0.024
TyG				
< 8.90	Ref		Ref	
8.90–9.30	1.23 (0.55–2.73)	0.592	0.40 (0.16–1.05)	0.060
≥ 9.30	2.27 (0.94–5.50)	0.067	0.58 (0.15–2.23)	0.404
TyG-WHtR				
< 5.55	Ref		Ref	
5.55–6.29	2.01 (0.94–4.31)	0.069	0.99 (0.17–5.79)	0.987
≥ 6.29	6.33 (3.31–12.08)	< 0.001	11.72 (2.65–51.92)	0.003
METS-IR				
< 46.27	Ref		Ref	
46.27–57.04	1.37 (0.70–2.67)	0.329	4.56 (1.51–13.78)	0.010
≥ 57.04	5.01 (2.63–9.55)	< 0.001	16.55 (4.00–68.38)	<.001
**Age ≥ 60 years (*n*** **= 397)**				
HOMA-IR				
< 3.11	Ref		Ref	
3.11–5.81	3.73 (1.07–13.06)	0.041	1.46 (0.53–4.08)	0.440
≥ 5.81	12.30 (4.33–34.92)	< 0.001	3.36 (0.97–11.65)	0.055
TyG				
< 8.90	Ref		Ref	
8.90–9.30	1.09 (0.45–2.65)	0.835	1.12 (0.31–4.05)	0.854
≥ 9.30	2.43 (0.86–6.92)	0.089	0.84 (0.30–2.36)	0.726
TyG-WHtR				
< 5.55	Ref		Ref	
5.55–6.29	3.50 (1.59–7.72)	0.004	1.96 (0.59–6.52)	0.250
≥ 6.29	6.38 (2.88–14.14)	< 0.001	6.47 (2.14–19.60)	0.003
METS-IR				
< 46.27	Ref		Ref	
46.27–57.04	2.20 (0.94–5.19)	0.068	0.97 (0.29–3.29)	0.958
≥ 57.04	9.30 (4.45–19.46)	< 0.001	3.58 (1.38–9.25)	0.012
**Gender-male (*n*** **= 407)**				
HOMA-IR				
< 3.11	Ref		Ref	
3.11–5.81	3.36 (1.31–8.61)	0.015	3.15 (0.95–10.41)	0.059
≥ 5.81	8.74 (3.15–24.19)	< 0.001	4.62 (1.75–12.23)	0.004
TyG				
< 8.90	Ref		Ref	
8.90–9.30	1.14 (0.33–3.89)	0.829	0.96 (0.32–2.83)	0.930
≥ 9.30	1.96 (0.64–5.96)	0.218	1.00 (0.24–4.23)	0.999
TyG-WHtR				
< 5.55	Ref		Ref	
5.55–6.29	2.87 (1.58–5.22)	0.002	1.47 (0.30–7.23)	0.614
≥ 6.29	23.36 (9.59–56.87)	< 0.001	11.14 (2.05–60.51)	0.008
METS-IR				
< 46.27	Ref		Ref	
46.27–57.04	2.41 (0.84–6.89)	0.095	0.51 (0.20–1.34)	0.159
≥ 57.04	11.42 (3.87–33.70)	< 0.001	4.47 (1.49–13.41)	0.011
**Gender-female (*n*** **= 469)**				
HOMA-IR				
< 3.11	Ref		Ref	
3.11–5.81	1.97 (0.91–4.25)	0.079	2.89 (0.57–14.69)	0.185
≥ 5.81	3.61 (1.56–8.36)	0.005	4.92 (1.18–20.45)	0.031
TyG				
< 8.90	Ref		Ref	
8.90–9.30	1.22 (0.59–2.54)	0.568	0.41 (0.15–1.12)	0.077
≥ 9.30	2.94 (1.54–5.62)	0.003	0.60 (0.16–2.20)	0.415
TyG-WHtR				
< 5.55	Ref		Ref	
5.55–6.29	2.09 (0.94–4.64)	0.068	1.31 (0.24–7.03)	0.737
≥ 6.29	4.88 (2.33–10.21)	< 0.001	8.78 (2.02–38.27)	0.007
METS-IR				
< 46.27	Ref		Ref	
46.27–57.04	0.94 (0.42–2.15)	0.885	3.40 (1.20–9.58)	0.024
≥ 57.04	3.82 (2.12–6.90)	< 0.001	7.96 (2.27–27.91)	0.003

*METS-IR* metabolic score for insulin resistance, *TyG* triglyceride/glucose, *HOMA-IR* homeostasis model assessment of IR, *TyG-WHtR* triglyceride glucose-waist-to-height ratio, *OR* odds ratio, *CI* confidence interval

Crude model: confounding variables were not adjusted

^a^Adjusted Model: education level, hepatitis, cardiovascular disease (CVD), alanine aminotransferase (ALT), drug for hypertension, and protein were adjusted

^b^Adjusted Model: family income-to-poverty ratio, diastolic blood pressure, CVD, ALT, aspartate aminotransferase, gamma glutamyl transferase, platelet count, albumin, drug for diabetes, antiviral agents, and hepatic steatosis were adjusted

Furthermore, the statistical significance of the relationship between HOMA-IR, TyG-WHtR, and METS-IR with hepatic fibrosis in different populations is evident. However, there is no statistically significant association between HOMA-IR and liver fibrosis among individuals aged 60 years or older.

## Discussion

Our study investigated the association between four indicators reflecting IR and hepatic steatosis and fibrosis in patients with MetS using nationally representative data. The findings found that HOMA-IR, TyG-WHtR, and METS-IR were associated with an increased odd of hepatic steatosis and fibrosis in patients with MetS. Moreover, we also noticed an association between the TyG index and hepatic steatosis. The results from ROC curve analyses indicated that TyG-WHtR had good diagnostic values for predicting the risk of hepatic steatosis and fibrosis among patients with MetS.

The etiology of NAFLD remains poorly elucidated, but the contribution of IR to the progression of NAFLD has been extensively acknowledged. The current clinical applicability of hyperinsulinemic-euglycemic glucose clamp, considered as the gold standard for evaluating IR, is limited due to its time-consuming nature and high expenses [22]. In the recent years, certain indicators have also been proven to be dependable surrogate markers of IR, including HOMA-IR, TyG index, TyG-WHtR, and METS-IR. Evidence has suggested that these indicators were related to risk of hepatic steatosis and fibrosis, and could serve as predictive markers for this condition. A cohort study conducted in Japanese populations have displayed that the presence of TyG index is linked to the occurrence of NAFLD [23]. In the study of Gutierrez et al., HOMA-IR demonstrates an independent correlation with the occurrence of NAFLD in adult patients with type 2 diabetes (T2DM), suggesting its potential utility as a diagnostic tool for identifying this condition in clinical settings [24]. A recent study conducted on patients with T2DM found that combining TyG index and obesity parameters index (TyG-WHtR) was more effective than using TyG index alone in identifying hepatic steatosis [25]. This highlights the potential of TyG-WHtR as a straightforward and efficient marker for screening fatty liver disease in patients with T2DM. Until now, there has been a lack of extensive investigation into the correlation between HOMA-IR, TyG index, TyG-WHtR, and METS-IR, and the risk of hepatic steatosis and fibrosis among patients with MetS.

In the present study, we included 876 patients with MetS, to evaluate and compare the diagnostic value of four parameters (HOMA-IR, TyG index, TyG-WHtR, and METS-IR) on the risk of hepatic steatosis and fibrosis. The results observed that HOMA-IR, TyG-WHtR, and METS-IR were associated with hepatic steatosis and fibrosis of patients with MetS. Further subgroup analyses also supported these conclusions. Also, METS-IR, HOMA-IR and TyG-WHtR also were found to be related to severe hepatic steatosis. It is worth mentioning that TyG index was found in this study was only linked with hepatic steatosis, and there was no statistical difference in the relationship between TyG index and hepatic fibrosis, which was inconsistent with the results of a previous study. Guo’ study indicated that the TyG index exhibits a positive correlation with the severity of hepatic steatosis and the existence of hepatic fibrosis in Chinese population with NAFLD [26]. Possible factors may be the sources of the sample size. In addition, the study primarily focuses on individuals with MetS, and there may exist variations in IR levels. Moreover, overweight and obesity, particularly central obesity, are integral components of MetS, which could potentially explain why TYG-WHtR and METS-IR exhibit superior performance compared to TyG. Further prospective investigations are required to authenticate the findings of this study. In addition, the analysis of ROC curves revealed that TyG-WHTR demonstrated a higher predictive value for hepatic steatosis (AUC: 0.705, 95%CI: 0.668–0.743) and hepatic fibrosis (AUC: 0.703, 95%CI: 0.655–0.751) in patients with MetS compared to the other three indicators. In summary, the results of this investigation indicate that TyG-WHtR, being an inexpensive and easily accessible indicator, has the potential to facilitate early intervention in managing hepatic steatosis and fibrosis.

The primary advantage of this study lies in the precise identification of liver steatosis and fibrosis through the application of transient elastography during liver ultrasound, yielding exceptional levels of accuracy, which provided significant epidemiological evidence for the relationship between four IR indicators and hepatic steatosis and fibrosis in patients with MetS. Some limitations should be noted. First, this is a cross-sectional study, we could not establish a causal relationship of four IR indicators and hepatic steatosis and fibrosis. Second, this study was conducted exclusively on U.S. population, and it is crucial to corroborate our results among heterogeneous populations. Furthermore, this study was limited by the database (NHANES 2017–2018) and the sample size was limited. Larger-size and multi-center studies should be performed in future.

## Conclusion

Our study demonstrated that HOMA-IR, TyG-WHtR, and METS-IR may be associated with the risk of hepatic steatosis and fibrosis among the U.S. adult population with MetS. In addition, TyG-WHtR may have a good predictive value for hepatic steatosis and hepatic fibrosis.

### Supplementary Information

**Additional file 1.**

## Data Availability

The datasets generated and/or analyzed during the current study are available in the NHANES database, https://wwwn.cdc.gov/nchs/nhanes/.

## Abbreviations

NAFLD: Non-alcoholic fatty liver disease
MetS: Metabolic syndrome
IR: Insulin resistance
HOMA-IR: Homeostasis model assessment of IR
TyG: Triglyceride/glucose
TyG-WHtR: Triglyceride glucose-waist-to-height ratio
METS-IR: Metabolic score for IR
NHANES: National Health and Nutrition Examination Survey
WHtR: Waist-to-height ratio
BMI: Body mass index
VCTE: Vibration Controlled Transient Elastography
M: Medium
XL: Extra-large
CAP: Controlled attenuation parameter
LSM: Liver stiffness measurement
PIR: Income-to-poverty ratio
SBP: Systolic blood pressure
DBP: Diastolic blood pressure
CKD: Chronic kidney disease
CVD: Cardiovascular disease
ALP: Alkaline phosphatase
GGT: Gamma glutamyl transferase
TC: Total cholesterol
LDL-C: Low-density lipoprotein cholesterol
OR: Odds ratio
CI: Confidence interval
ROC: Receiver operating characteristics curve
