# Supplementary material for: Association of insulin resistance indicators with hepatic steatosis and fibrosis in patients with metabolic syndrome
Source: BMC Gastroenterol. 2024 Jan 9;24:26. doi: 10.1186/s12876-023-03095-6 (PMC10775571; doi:10.1186/s12876-023-03095-6)
Supplement: Supplementary file 1 — Additional file 1. [file 12876_2023_3095_MOESM1_ESM.docx]

**Supplemental Table 1 Sensitivity analysis of before and after interpolation**

| **Variables** | **Before interpolation** | **After interpolation** | ***P*** |
| --- | --- | --- | --- |
| Family PIR, Mean (S.E) | 2.81 (0.10) | 2.80 (0.10) | 0.275 |
| SBP, Mean (S.E) | 131.25 (0.67) | 131.37 (0.63) | 0.112 |
| DBP, Mean (S.E) | 76.13 (0.64) | 76.09 (0.64) | 0.178 |
| LDL-C, mg/dL, Mean (S.E) | 114.55 (2.35) | 114.49 (2.57) | 0.912 |
| Energy, Mean (S.E) | 2227.94 (51.55) | 2220.76 (50.98) | 0.230 |
| Carbohydrate, Mean (S.E) | 259.61 (6.24) | 258.88 (6.21) | 0.315 |
| Protein, Mean (S.E) | 83.77 (2.07) | 83.54 (2.04) | 0.349 |
| Total fat, Mean (S.E) | 91.49 (2.49) | 91.19 (2.41) | 0.197 |
| Vitamin E, Mean (S.E) | 0.68 (0.19) | 0.67 (0.18) | 0.511 |

PIR=income-to-poverty ratio; SBP=systolic blood pressure; DBP=diastolic blood pressure; LDL-C=low-density lipoprotein cholesterol.

**Supplemental Table 2 Selection of covariates by weighted univariate logistic regression analysis**

| Variables | Hepatic steatosis | | Hepatic fibrosis | |
| --- | --- | --- | --- | --- |
|  | OR (95%CI) | *P* | OR (95%CI) | *P* |
| Age, years | 1.00 (0.99-1.02) | 0.535 | 1.01 (0.99-1.02) | 0.388 |
| Gender |  |  |  |  |
| Male | Ref |  | Ref |  |
| Female | 0.71 (0.44-1.12) | 0.132 | 1.14 (0.56-2.31) | 0.692 |
| Race |  |  |  |  |
| Mexican American | Ref |  | Ref |  |
| Other Hispanic | 0.64 (0.29-1.39) | 0.237 | 1.40 (0.55-3.56) | 0.459 |
| Non-Hispanic White | 0.60 (0.28-1.30) | 0.178 | 0.86 (0.41-1.83) | 0.684 |
| Non-Hispanic Black | 0.49 (0.24-1.01) | 0.052 | 0.66 (0.34-1.27) | 0.195 |
| Other Race | 0.52 (0.22-1.23) | 0.127 | 0.80 (0.37-1.74) | 0.551 |
| Education level |  |  |  |  |
| High school degree or less | Ref |  | Ref |  |
| High school education | 2.89 (1.22-6.87) | 0.019 | 1.56 (0.79-3.05) | 0.182 |
| High school degree or above | 1.60 (0.92-2.79) | 0.091 | 1.08 (0.58-1.99) | 0.797 |
| Unknown | 1.34 (0.28-6.38) | 0.692 | 2.99 (0.74-12.02) | 0.114 |
| Family PIR | 1.02 (0.85-1.23) | 0.791 | 0.85 (0.72-0.99) | 0.040 |
| Drinking status |  |  |  |  |
| No | Ref |  | Ref |  |
| Yes | 1.06 (0.50-2.25) | 0.872 | 0.47 (0.22-1.02) | 0.056 |
| Smoking status |  |  |  |  |
| Never smoked | Ref |  | Ref |  |
| Used to smoke and now quit | 0.96 (0.58-1.60) | 0.863 | 1.35 (0.66-2.77) | 0.384 |
| Still smoking | 0.87 (0.49-1.55) | 0.615 | 1.04 (0.55-1.95) | 0.906 |
| Physical activity, n (%) |  |  |  |  |
| ≤750 MET⸳ min | Ref |  | Ref |  |
| >750 MET⸳ min | 1.10 (0.65-1.84) | 0.714 | 0.57 (0.28-1.14) | 0.104 |
| SBP | 1.01 (0.99-1.03) | 0.305 | 1.00 (0.99-1.01) | 0.974 |
| DBP | 1.00 (0.98-1.01) | 0.700 | 0.97 (0.96-0.99) | 0.016 |
| Hepatitis |  |  |  |  |
| No | Ref |  | Ref |  |
| Yes | 0.37 (0.14-0.97) | 0.044 | 1.76 (0.48-6.50) | 0.369 |
| CKD |  |  |  |  |
| No | Ref |  | Ref |  |
| Yes | 0.80 (0.27-2.33) | 0.659 | 1.90 (0.90-4.00) | 0.087 |
| CVD |  |  |  |  |
| No | Ref |  | Ref |  |
| Yes | 1.89 (1.08-3.31) | 0.028 | 2.20 (1.11-4.37) | 0.027 |
| ALT, U/L | 1.02 (1.01-1.04) | 0.023 | 1.02 (1.01-1.04) | 0.006 |
| AST, U/L) | 1.01 (0.98-1.04) | 0.522 | 1.04 (1.01-1.06) | 0.003 |
| ALP, IU/L | 1.00 (0.99-1.01) | 0.482 | 1.01 (0.99-1.02) | 0.270 |
| GGT, IU/L | 1.00 (1.00-1.01) | 0.343 | 1.01 (1.01-1.02) | 0.006 |
| TC, mg/dL | 1.00 (1.00-1.00) | 0.808 | 1.00 (0.99-1.00) | 0.183 |
| LDL-C, mg/dL | 1.00 (1.00-1.01) | 0.593 | 1.00 (0.99-1.00) | 0.304 |
| Hs-CRP, mg/L | 1.00 (0.97-1.03) | 0.979 | 1.01 (0.99-1.03) | 0.146 |
| Platelet count, 1000 cells/uL | 1.00 (1.00-1.00) | 0.281 | 0.99 (0.99-0.99) | 0.031 |
| Total bilirubin, umol/L | 1.01 (0.95-1.07) | 0.840 | 1.03 (1.00-1.07) | 0.080 |
| Serum creatinine, mg/dL | 0.87 (0.42-1.80) | 0.683 | 0.93 (0.31-2.78) | 0.894 |
| Urinary creatinine, mg/dL | 1.00 (1.00-1.01) | 0.117 | 1.00 (0.99-1.00) | 0.934 |
| Serum ALB, g/L | 1.02 (0.91-1.14) | 0.714 | 0.90 (0.84-0.96) | 0.004 |
| Urinary ALB, mg/L | 1.00 (1.00-1.00) | 0.801 | 1.00 (1.00-1.00) | 0.061 |
| Drug for diabetes, n (%) |  |  |  |  |
| No | Ref |  | Ref |  |
| Yes | 1.83 (1.01-3.33) | 0.050 | 2.77 (1.38-5.57) | 0.007 |
| Drug for hypertension, n (%) |  |  |  |  |
| No | Ref |  | Ref |  |
| Yes | 1.74 (1.06-2.86) | 0.030 | 1.48 (0.63-3.51) | 0.347 |
| Drug for dyslipidemia, n (%) |  |  |  |  |
| No | Ref |  | Ref |  |
| Yes | 1.46 (0.86-2.48) | 0.145 | 0.98 (0.55-1.74) | 0.935 |
| Antiviral agents, n (%) |  |  |  |  |
| No | Ref |  | Ref |  |
| Yes | 1.02 (0.05-20.76) | 0.986 | - | <.001 |
| Glucocorticoids, n (%) |  |  |  |  |
| No | Ref |  | Ref |  |
| Yes | 1.07 (0.36-3.17) | 0.900 | 0.21 (0.04-1.16) | 0.070 |
| Drug induce hepatic steatosis n (%) |  |  |  |  |
| No | Ref |  | Ref |  |
| Yes | 0.37 (0.08-1.62) | 0.169 | 1.46 (0.23-9.36) | 0.671 |
| Energy | 1.00 (1.00-1.00) | 0.189 | 1.00 (1.00-1.00) | 0.576 |
| Carbohydrate | 1.00 (1.00-1.00) | 0.404 | 1.00 (1.00-1.00) | 0.344 |
| Protein | 1.01 (1.01-1.01) | 0.026 | 1.00 (0.99-1.01) | 0.807 |
| Total fat | 1.00 (1.00-1.01) | 0.134 | 1.00 (1.00-1.00) | 0.972 |
| Vitamin E | 0.97 (0.91-1.04) | 0.382 | 0.98 (0.91-1.07) | 0.671 |
| Hepatic steatosis |  |  |  |  |
| No | - | - | Ref |  |
| Yes | - | - | 2.82 (1.45-5.47) | 0.005 |

PIR=income-to-poverty ratio; SBP=systolic blood pressure; DBP=diastolic blood pressure; CKD= chronic kidney disease; CVD=cardiovascular disease; ALT=alanine aminotransferase; AST= aspartate aminotransferase; ALP=alkaline phosphatase; GGT=gamma glutamyl transferase; TC= total cholesterol; LDL-C= low-density lipoprotein cholesterol; hs-CRP=high-sensitivity C-reactive protein; ALB=albumin; OR=odds ratio; CI=confidence interval.

**Supplemental Table 3 Relationship of four IR indicators and different degrees of hepatic steatosis.**

| **Indicators** | **Crude Model** | | **Adjusted Model** | |
| --- | --- | --- | --- | --- |
|  | **OR (95%CI)** | ***P*** | **OR (95%CI)** | ***P*** |
| HOMA_IR |  |  |  |  |
| <3.11 | Ref |  | Ref |  |
| 3.11-5.81 | 1.58 (0.69-3.62) | 0.253 | 1.41 (0.62-3.22) | 0.387 |
| ≥5.81 | 4.18 (1.61-10.82) | 0.006 | 3.12 (1.11-8.79) | 0.034 |
| TyG |  |  |  |  |
| <8.90 | Ref |  | Ref |  |
| 8.90-9.30 | 1.12 (0.52-2.44) | 0.757 | 1.21 (0.58-2.53) | 0.597 |
| ≥9.30 | 2.36 (0.94-5.94) | 0.065 | 1.97 (0.77-5.05) | 0.143 |
| TyG-WHtR |  |  |  |  |
| <5.55 | Ref |  | Ref |  |
| 5.55-6.29 | 2.10 (0.82-5.36) | 0.114 | 2.28 (0.88-5.88) | 0.083 |
| ≥6.29 | 4.43 (2.12-9.25) | <0.001 | 4.99 (2.35-10.59) | <0.001 |
| METS-IR |  |  |  |  |
| <46.27 | Ref |  | Ref |  |
| 46.27-57.04 | 2.75 (1.76-4.30) | <0.001 | 1.99 (1.25-3.19) | 0.007 |
| ≥57.04 | 6.57 (3.74-11.55) | <0.001 | 4.72 (2.75-8.10) | <0.001 |

METS-IR=metabolic score for insulin resistance; TyG=triglyceride/glucose; HOMA-IR=homeostasis model assessment of IR; TyG-WHtR=triglyceride glucose-waist-to-height ratio; OR=odds ratio; CI=confidence interval.

Crude model: confounding variables were not adjusted.

Adjusted Model: gender, family income-to-poverty ratio, smoke status, diastolic blood pressure, alanine aminotransferase, aspartate aminotransferase, gamma glutamyl transferase, hypersensitive C-reactive protein, energy, protein, and total fat.
